# Novel positive allosteric modulators of sigma-1 receptor

**DOI:** 10.1186/2193-1801-4-S1-P51

**Published:** 2015-06-12

**Authors:** Edijs Vavers, Liga Zvejniece, Grigory Veinberg, Baiba Svalbe, Ilona Domracheva, Reinis Vilskersts, Maija Dambrova

**Affiliations:** Latvian Institute of Organic Synthesis, Latvia; Riga Stradins University, Latvia

**Keywords:** Sigma-1 receptor, positive allosteric modulation, cognition

## Background:

The Sigma-1 receptor is a chaperone protein that modulates intracellular calcium signalling of the endoplasmatic reticulum and is involved in learning and memory processes. The aim of the present study was to compare in vitro Ca2+ concentration modulating activity and in vivo behavioural effects of enantiomers of methylphenylpiracetam, a novel positive allosteric modulator of Sigma-1 receptors.Methods: The activity of enantiomers was compared in the model of electrically stimulated rat vas deferens and bradykinin-induced intracellular Ca2+ concentration ([Ca2+]i) increase assay in neuroblastoma-glioma (NG-108) hybrid cells, PRE-084 was used as a selective Sigma-1 receptor agonist. Effects on contextual memory, locomotor activity and antidepressant activity were evaluated in ICR male mice in passive avoidance (PA) response, open field and Porsolt forced swimming tests, respectively. The rota-rod, traction and chimney tests were used to screen effects of the compounds on muscle function and coordination.

## Results:

Enantiomers with R-configuration at the C-4 chiral centre in the 2-pyrrolidone ring both significantly increased PRE-084 activity in vitro and almost two-fold increased the retention time in the PA test in vivo. The behavioural side effects were slightly more expressed for the enantiomers with S-configuration at the C-5 chiral centre. The enantiomers of methylphenylpiracetam did not induce any significant effects on the locomotor and depressive condition in the mice.

## Conclusions:

Pharmacological stimulation of sigma-1 receptor is an emerging approach for cognition enhancement. The R-configuration enantiomers of methylphenylpiracetam are more active positive allosteric modulators of Sigma-1 receptor than S-configuration enantiomers. The Sigma-1 receptor modulatory activity of compounds correlated with the memory enhancing effects.

## Acknowledgements

The study was supported by the Latvian Science Council grant No. 108/2013.

